# 

**Published:** 1907-07-15

**Authors:** 


CONTENTS

Event and Comment - - - - - - - - - 323

Michigan's New Dental Law - - - - - - - - 326

The Treatment of Putrescent Teeth, - *By L. P. Hall, D.DS.* 329

The Managemeut of a Dental Practice,
*By Edward C. Briggs, M.D., D.M.D.* 351

With Our Editors - - - - - - - - - 360

Indents - - - - - - - - - - - 368

Announcements - - - - - - - - - - 374

